# New Strategies for Treatment of Diabetic Macular Edema

**DOI:** 10.1155/2018/4292154

**Published:** 2018-08-19

**Authors:** Yoshihiro Takamura, Kishiko Ohkoshi, Toshinori Murata

**Affiliations:** ^1^Department of Ophthalmology, University of Fukui, Fukui, Japan; ^2^Department of Ophthalmology, St. Luke's International Hospital, Tokyo, Japan; ^3^Department of Ophthalmology, Shinshu University School of Medicine, Matsumoto, Japan

Diabetic macular edema (DME) is the most frequent cause of vision loss in patients with diabetes and is an important public health problem. Recent randomized clinical trials have shown anti-vascular endothelial growth factor (VEGF) therapy improved visual acuity and macular swelling, and currently it has become the first line of the treatment of DME. However, the pathogenesis of DME is multifactorial, and several therapeutic modalities have been proposed for the treatment of DME. New strategy with the use of not only anti-VEGF drugs but also corticosteroids, laser photocoagulation, and vitrectomy can be alternative therapies for the persistent or refractory to anti-VEGF drugs. This special issue was intended to serve as a platform for sharing current data and new innovations in the management of DME.

Anti-VEGF drugs have become the gold standard for the treatment of DME, replacing macular laser photocoagulation. Best et al. showed the real-life efficacy of ranibizumab in DME at 12 months and the need for a large number of injections to achieve better visual outcomes. They also showed a trend to a lower compliance in diabetic versus neovascular age-related macular degeneration (nAMD) patients: only 16.8% of nAMD patients were lost to follow-up at one year versus 25.45% in diabetic patients. Many eyes respond well to anti-VEGF agents; nevertheless, some do not achieve favorable edema control, and these cases are referred to as refractory DME. Switching from one anti-VEGF drug to another is a viable first step for resistant DME management. Demircan et al. compared a switch group that comprised patients who were switched to aflibercept after showing a poor response to previous ranibizumab treatment with a ranibizumab group composed of patients who continued with ranibizumab injections despite the presence of poor response to this treatment. They showed that the switching therapy from intravitreal ranibizumab to aflibercept in persistent DME provided only morphologic improvement. The discrepancy between morphologic and functional outcomes may be explained by irreversible functional damage caused by long-standing DME.

Anti-VEGF treatment requires repeated intravitreous injections to maintain the therapeutic effect, and safety concerns regarding long-term systemic suppression of VEGF, which may increase a serious risk of cerebrovascular accidents, are emerging. Especially, type 2 diabetic patients with DME or PDR were associated with a 2-fold higher risk of fatal cardiovascular accidents compared with those without DME or PDR. Thus, a new optical treatment modality should be developed to improve the cost-effectiveness, safety, and visual outcomes. Predominantly focal leakage from microaneurysms (MAs) showed less response to anti-VEGF therapy. Focal laser treatment leads to the occlusion of MAs, pathologic vessels, or subretinal sites of leakage. The navigated laser photocoagulator has an eye-tracking laser delivery system and allows more accuracy for focal laser photocoagulation than conventional focal laser therapy for DME. Kato et al. showed that focal photocoagulation using Navilas 577+ aiming MAs, mainly localized outside of the perifoveal capillary network, was effective in treating DME with improvement in macular edema on OCT over 6 months. The navigated photocoagulation seems to demonstrate a higher laser spot application accuracy in focal laser therapy of DME than conventional laser technique. In their case series, indocyanine green angiography (ICGA) guide navigated laser was performed to most of the study eyes (84%). Indocyanine green dye is 98% bound to lipoproteins in the blood. Thus, the dye hardly leaks, and ICGA defines the detailed retinal vascular abnormalities better than fluorescein angiography.

In addition to the accuracy, the less invasion of laser ablation in the retinal tissue is also clinically important. Although panretinal photocoagulation (PRP) is the standard therapy to inhibit the progression of diabetic retinopathy, PRP sometimes results in the worsening of macular edema. Recently developed short-pulse laser treatment is quicker, generates less heat, and is less painful to eyes than the conventional laser. Moreover, short-pulse laser treatment induces less inflammation, fewer up-regulation of inflammatory cytokines after PRP, and less macular thickening in patients with diabetic retinopathy than the conventional pulse duration. Higaki et al. demonstrated that fundus autofluorescence (FAF) images were useful to evaluate the changes in the photocoagulation scar sizes. The scars with the short-pulse laser showed lower expansion rates than those of the conventional laser. Analysis of FAF is an effective method to observe the functions of the retinal pigment epithelial (RPE) cells. Since retinal laser photocoagulation targets RPE, FAF analysis after laser photocoagulation may be an effective method to evaluate the RPE alterations and efficacy of laser photocoagulation.

Pars plana vitrectomy (PPV) is alternative strategy as a treatment for refractory DME. In the case of vitreoretinal interface abnormality, PPV can relieve the tractional component and can result in resolution of the edema. Vitrectomy may also contribute to a more efficient clearance of VEGF and other cytokines and better oxygen access from the anterior segment to the retina, thereby reducing DME. Hadi et al. showed the efficacy of subretinal balanced salt solution (BSS) injections in conjunction with conventional vitrectomy. Vitrectomy with the planned foveal detachment technique appears to be a promising solution for DME resistant to more than one anti-VEGF agent, intravitreal corticosteroids. The adjunctive therapy in the combination of the drugs with vitrectomy is also considered as a useful tool. Cui et al. compared the effect and safety of intravitreal injection of conbercept (IVC), ranibizumab (IVR), or triamcinolone acetonide (IVTA) on 23 gauge pars plana vitrectomy (PPV) for proliferative diabetic retinopathy. They showed that IVC and IVR could reduce the difficulty of the operation and improve the success rate of the surgery. In IVC and IVR groups, the fibrous membranes were easily separated from the retina with an individual of bleeding. Compared with IVTA group, IVC and IVR groups had more visual acuity gains after surgeries.

The guest editors appreciate the all authors of the papers submitted to this special issue. The editors also would thank the all reviewers, who devoted their energy and time and whose insightful comments and suggestions helped improve the manuscripts selected for this special issue. We hope that the readers of this special issue will find its contents interesting and clinically valuable.

*Yoshihiro Takamura* *Kishiko Ohkoshi* *Toshinori Murata*

